# Timing of Enteral Feeding in Cerebral Malaria in Resource-Poor Settings: A Randomized Trial

**DOI:** 10.1371/journal.pone.0027273

**Published:** 2011-11-16

**Authors:** Richard J. Maude, Gofranul Hoque, Mahtab Uddin Hasan, Abu Sayeed, Shahena Akter, Rasheda Samad, Badrul Alam, Emran Bin Yunus, Ridwanur Rahman, Waliur Rahman, Romal Chowdhury, Tapan Seal, Prakaykaew Charunwatthana, Christina C. Chang, Nicholas J. White, M. Abul Faiz, Nicholas P. J. Day, Arjen M. Dondorp, Amir Hossain

**Affiliations:** 1 Mahidol-Oxford Tropical Medicine Research Unit, Faculty of Tropical Medicine, Mahidol University, Bangkok, Thailand; 2 Centre for Tropical Medicine, Nuffield Department of Clinical Medicine, John Radcliffe Hospital, University of Oxford, Oxford, United Kingdom; 3 Chittagong Medical College Hospital, Chittagong, Bangladesh; 4 Fatik Chari Thana Health Complex, Chittagong, Bangladesh; 5 Hossain Shahid Sohrawardy Medical College, Dhaka, Bangladesh; 6 Sir Salimullah Medical College, Dhaka, Bangladesh; Laboratory of Malaria Immunology and Vaccinology, United States of America

## Abstract

**Background:**

Early start of enteral feeding is an established treatment strategy in intubated patients in intensive care since it reduces invasive bacterial infections and length of hospital stay. There is equipoise whether early enteral feeding is also beneficial in non-intubated patients with cerebral malaria in resource poor settings. We hypothesized that the risk of aspiration pneumonia might outweigh the potential benefits of earlier recovery and prevention of hypoglycaemia.

**Method and Findings:**

A randomized trial of early (day of admission) versus late (after 60 hours in adults or 36 hours in children) start of enteral feeding was undertaken in patients with cerebral malaria in Chittagong, Bangladesh from May 2008 to August 2009. The primary outcome measures were incidence of aspiration pneumonia, hypoglycaemia and coma recovery time. The trial was terminated after inclusion of 56 patients because of a high incidence of aspiration pneumonia in the early feeding group (9/27 (33%)), compared to the late feeding group (0/29 (0%)), p = 0.001). One patient in the late feeding group, and none in the early group, had hypoglycaemia during admission. There was no significant difference in overall mortality (9/27 (33%) vs 6/29 (21%), p = 0.370), but mortality was 5/9 (56%) in patients with aspiration pneumonia.

**Conclusions:**

In conclusion, early start of enteral feeding is detrimental in non-intubated patients with cerebral malaria in many resource-poor settings. Evidence gathered in resource rich settings is not necessarily transferable to resource-poor settings.

**Trial Registration:**

Controlled-Trials.com ISRCTN57488577

## Introduction

Even with optimal antimalarial treatment with parenteral artesunate, mortality of severe malaria remains 15% in adults and 9% in children [1], [2]. Improvements in supportive care have the potential to lower this still high case fatality rate. However, in resource poor settings more technically demanding interventions, such as mechanical ventilation and renal replacement therapy, will only be feasible in a minority of patients. Other treatments, such as enteral feeding via a nasogastric tube, are easier to achieve.

In the well equipped intensive care setting early start of enteral feeding is now common practice in a wide variety of patients, including those with sepsis [3]. Nutrition supplies vital cell substrates, antioxidants, vitamins, and minerals, all essential for normal cell function. Studies have shown that early enteral feeding preserves the barrier function of the gut, has positive effects on the hepatosplanchnic circulation and immune functions, is associated with a decrease in hypermetabolism and organ failure, and reduces the incidence of bacteraemia [4], [5], [6], [7], [8], [9]. A meta-analysis of 15 studies in 753 critically ill surgical patients concluded that start of enteral feeding within 36 hours of admission reduced the incidence of invasive infections, and reduced the length of hospital stay and costs [3]. Patients with severe malaria are often poorly nourished and hypoglycaemia is a common complication, especially in those receiving quinine [10]. Bacteraemia is more common in severe malaria, in particular with non-typhoid Salmonella species [11] and thought to be related to microvascular obstruction in the gut causing increased bacterial translocation. Early start of enteral feeding has been shown to restore gut barrier function [12]. Studies on enteral feeding in the intensive care setting are performed in patients with an airway protected by endotracheal intubation. Although this does not prevent aspiration pneumonia [13], aspiration pneumonia is a rare complication, especially with the use of post-pyloric enteral feeding tubes [14], [15], [16], [17], [18] and avoidance of supine positioning of patients by elevating the head of the bed by at least 30^o^ [18], [19]. Routine endotracheal intubation in comatose patients with malaria is not a feasible option in resource poor tropical countries where malaria is endemic. The use of enteral feeding through a nasogastric (NG) tube might thus induce a significant risk of aspiration pneumonia, outweighing the theoretical benefits.

We therefore performed an open randomized trial at a tertiary referral hospital in Chittagong, Bangladesh to compare early versus late start of nasogastric tube feeding in patients with cerebral malaria. Incidences of aspiration pneumonia, hypoglycaemia and coma recovery time were the primary outcome measures.

## Methods

The protocol for this trial and supporting CONSORT checklist are available as supporting information; see Checklist S1 and Protocol S1.

### Ethics statement

This study was conducted according to the principles expressed in the Declaration of Helsinki. Ethical clearance for the study was obtained from the Ethical Review Committee of Chittagong Medical College, and from the Oxford Tropical Research Ethics Committee (OXTREC). ISRCTN registration number ISRCTN57488577. For all participants, prior written informed consent was obtained from an attending relative.

### Study site

The study was conducted at Chittagong Medical College Hospital (CMCH), Chittagong, Bangladesh from June 2008 to August 2009. CMCH is a 1000-bed teaching hospital with limited facilities for intensive care, oxygen therapy, blood transfusion, and renal dialysis. Provision of optimal nursing care is sometimes problematic because of the large patient load. Malaria transmission around Chittagong is seasonal and of low intensity.

### Patients

Adults and children ≥2 years with slide proven cerebral Plasmodium falciparum malaria (defined as a GCS<11or BCS <3 for pre-verbal children) were included in the study, provided that written informed consent was obtained. Patients with features of aspiration pneumonia on admission, based on an abnormal chest examination and pulse oximetry and/or chest x-ray findings were excluded. Other exclusion criteria were: pregnancy, diabetes mellitus requiring insulin or contraindications to enteral feeding (circulatory shock, mechanical bowel obstruction, ileus, ischaemic colitis, severe diarrhoea (>6 times per 24 hours), severe vomiting (>6 times per 24 hours) or severe dehydration, pancreatitis (laboratory confirmed: serum amylase >500 U/L), or children with severe malnutrition (according to WHO criteria)) [20].

### Procedures

On enrolment, a full history and physical examination were performed. Nutritional status of the patient was assessed by skin-fold thickness in the triceps region of the left arm, mid upper arm circumference, weight, height and presence of bilateral pedal oedema.

Patients were managed in accordance with the World Health Organization guidelines for malaria 2006 [21]. Antimalarial treatment was with intravenous artesunate (Guilin Pharmaceutical Co, Guangxi, China) followed by a full course of artemether-lumefantrine (Coartem^R^) when the patient had recovered sufficiently to eat and take tablets.

All patients received a nasogastric (NG) tube on admission. Patients were randomized in advance in blocks of 20 by an independent investigator to either receive enteral feeding upon admission through the NG tube (‘early feeding’*)*, or no enteral feeding until able to take oral food or up to a maximum starvation period of 60 hours in adults and children >12 years old and 36 hours in children ≤12 years old (‘late feeding’*)*. The randomization code was generated by a statistician at the Mahidol-Oxford Research Unit in Bangkok. Treatment allocations were kept in consecutively numbered sealed envelopes and opened by the research physician upon enrollment of the patient into the study.

The feed formula used was Renovit or Revit-R (for patients with renal insufficiency) (Fasska SA, Louvain la Neuve, Belgium). This was dissolved in bottled water according to the instructions of the manufacturer providing 1 kcal per ml feed.

The position of the NG tube was checked by a study doctor before starting the first feed by injecting air with a large syringe and listening over the abdomen, and before subsequent feeds by patient attendants who had been specifically trained by the study doctor by checking the position of a mark drawn on the tube at the time of insertion and by aspiration of gastric contents. The head of the bed was elevated to 30°–45°, where possible throughout the admission, otherwise for a minimum of 30 minutes after feeding followed by placing the patient in the recovery position. NG feeding for adults was started at a rate of 2–4 ml/kg feed given as a slow bolus every 2 hours with 10 feeds per day, omitting 2 late night feeds corresponding to delivery of 1000–2000 kCal per 24 hours to an adult weighing 50 kg. For children, the volume of enteral feeding was calculated by subtracting the amount given intravenously from the total amount of fluids required per 24 hours, based on age and body weight: (2–4years: 100–120ml/kg/day; 4–8years: 90–100ml/kg/day; 8–12years: 70–90; over 12 years: 60–70ml/kg/day). The total amount of enteral feed in children was divided into 12 feeds, given every 2 hours round-the-clock.

The volume of aspirated gastric content was measured 2-hourly just before every feed. Gastric retention was defined as >4 ml/kg in adults and >2/3^rd^ of the volume of the previous feed in children. If gastric retention was present, feeding was withheld and a prokinetic drug was administered (domperidone suppositories 20mg every 8 hours for adults and 15mg (1/2 suppository up to 5 years & 1 suppository over 5 years of age) for children). If gastric retention was not present, any aspirated feed in the syringe was discarded and feeds were continued every 2 hours according to schedule.

Adult patients (>12 years) in the late feeding group received 50–60 ml 5% dextrose-saline/kg/24 hours intravenously with an energy content of around 200 kCal/L, corresponding to 10–12 kCal/kg/24 hours. Paediatric patients (≤12 years) in the late feeding group received intravenous 10% dextrose in 0.45% saline in the volumes stated above until feeding was started. This contained 400 kCal/L corresponding to: 40–48 kCal/kg/24 hours for those aged 2–4 years, 36–40 kCal/kg/24 hours for 4–8 year olds and 24–28 kCal/kg/24 hours for 8–12 year olds.

As in many hospitals in the tropics, very few nursing staff were available (around 5 nurses for up to 100 patients) and much of the basic nursing care was provided by family members. The administration of enteral feed was performed largely by family members, with the support of the nursing staff and study doctors. Family members were instructed in the techniques for checking NG tube position using a pre-drawn mark, checking for gastric retention and giving NG feeds.

Patients were monitored 4-hourly by a study doctor for GCS or BCS, blood pressure, respiratory rate, temperature, oxygen saturation by pulse-oximetry, evidence of aspiration and signs of aspiration pneumonia. Patients were nursed in a 30^o^–45^o^ head tilt position whenever possible and turned on their other side every 2 hours. Fluid input and output was closely monitored. If needed in the comatose patients, maintenance of an open airway was secured with a Guedel.

On admission a venous blood sample was taken for parasitaemia, complete blood count, pH, bicarbonate, blood standardized base excess, glucose, electrolytes and blood urea nitrogen. Peripheral blood parasitaemia in thin or thick film, as appropriate, was assessed on admission, followed by 12 hourly sampling until complete parasite clearance, defined by 2 consecutive negative peripheral blood slides. Capillary blood glucose was checked 4 hourly until the patient regained consciousness. A blood glucose level ≤2.8 mmol/l was treated with 10% intravenous glucose (4 ml/kg body weight). Repeat blood count and biochemistry and blood cultures were done when indicated.

### Aspiration pneumonia

Aspiration pneumonia was diagnosed clinically from history and chest examination (fever, respiratory distress, auscultatory crackles and low oxygen saturation), and confirmed by chest X-ray (CXR). All patients had a CXR upon admission. The CXR was repeated upon indication and 3 days after the first one for follow-up. Treatment of aspiration pneumonia was with antibiotics (intravenous ceftriaxone plus metronidazole). Oxygen was given by nasal cannula or face mask where indicated. In the case of severe respiratory insufficiency, the patient was referred to the intensive care unit of CMCH for mechanical ventilation if a bed was available.

### Analysis

The primary outcome measures were the incidence of aspiration pneumonia, hypoglycaemia (<2.8 mmol/L) and coma recovery time (defined as the time to a Glasgow Coma Scale of 15/15 or BCS 5/5 in preverbal children). Incidence of sepsis was a secondary outcome measure. Sepsis was defined as the presence or suspicion of infection (other than malaria) plus systemic inflammatory response syndrome (SIRS) as indicated by ≥3 of the following criteria: prolonged fever i.e. axillary temperature ≥38°C or core temperature of ≤36°C; heart rate of ≥90 beats/min; a respiratory rate of ≥20 breaths/min (up to 5yrs ≥40) or the use of mechanical ventilation for an acute respiratory process; a white-cell count of ≥12×10^9^/l or ≤4×10^9^/l, or a differential count showing >10% immature neutrophils. Other secondary outcome measures were the time to sit independently, time to speak, time to eat independently, total duration (days) of admission in the hospital and in-hospital mortality.

A sample size of 62 in each treatment group was calculated in order to detect an increase in incidence in aspiration pneumonia from 10% to 30% with alpha of 0.05 and power of 0.8.

Statistical analysis was performed by SPSS software (version 15.0) and STATA (version 10). When appropriate, data were log transformed to obtain a normal distribution. Normally distributed data were compared using Student’s t test. The Mann-Whitney U test was used for nonpaired nonparametric data. Categorical data were compared by Pearson’s chi-squared test or Fisher’s exact test, as appropriate. The level of significance was p<0.05.

### Data safety monitoring

Adverse events were reported to an independent data safety monitoring committee (Chair: Prof. F. Nosten; report available on request). An interim analysis was planned and done after enrollment of 50 patients. Prespecified stopping rules for this interim analysis were a statistically significant difference between the groups in aspiration pneumonia, hypoglycaemia or mortality. On advice of the committee the trial was stopped on 2nd September 2009, because of the high incidence of aspiration pneumonia in the study group receiving early enteral feeding.

The study was externally monitored to assure compliance with the International Conference on Harmonisation of Good Clinical Practice guidelines.

## Results

**Figure 1:**
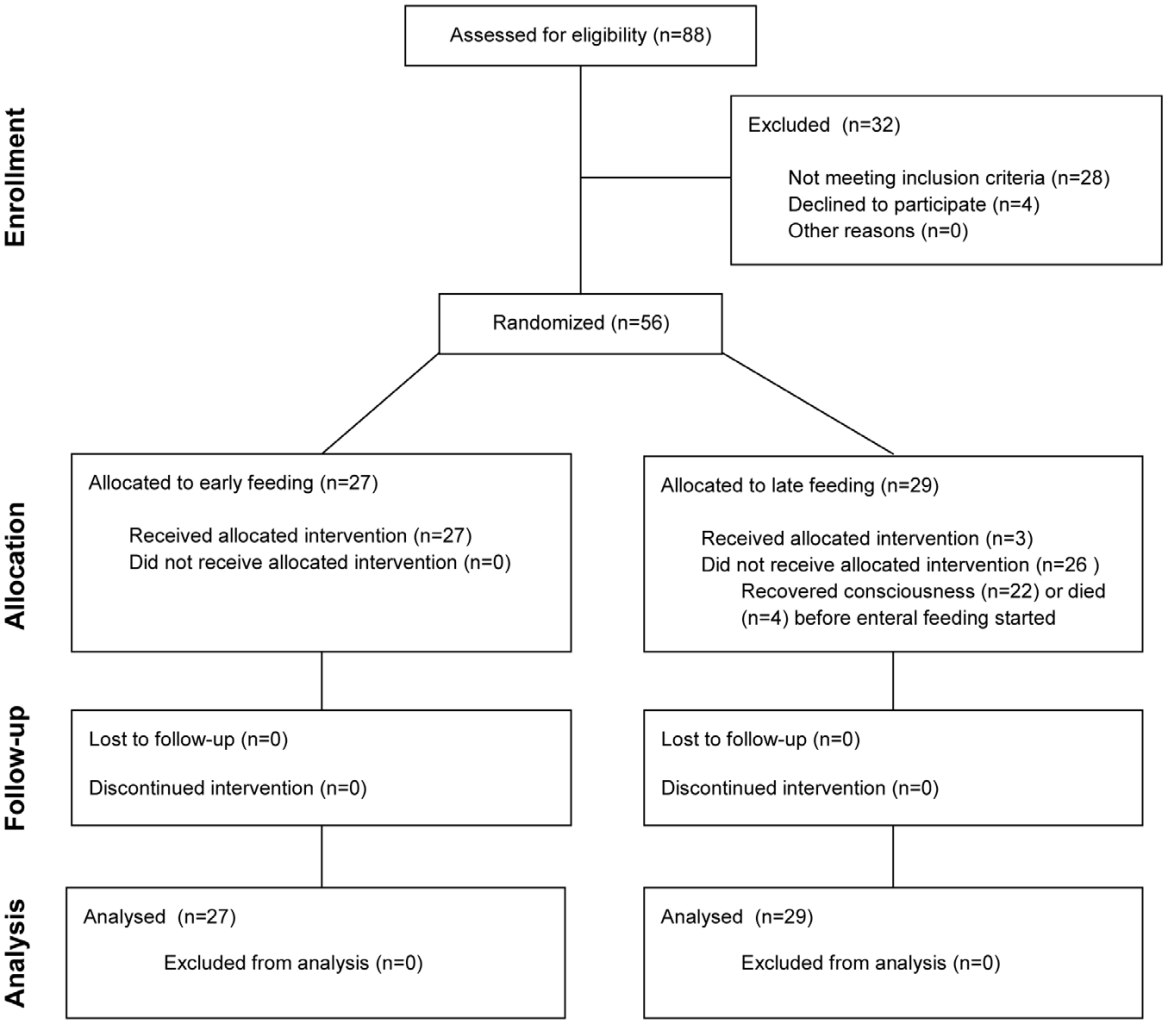
Consort flow diagram of the study.

A total of fifty six patients with cerebral malaria were enrolled and randomized to receive early (n = 27) or late (n = 29) start of enteral feeding (figure 1). Of these, 1/27 in the early and 3/29 in the late group were <14 years old. All patients in the early feeding group and 3/29 (10%) patients in the late feeding group received nasogastric feeding (figure 1). Most patients (n = 22) in the late feeding group recovered consciousness before 60 hours (>12 years) or 36 hours (≤12 years) after enrollment and could start oral feeding; another 4 patients died before reaching these cut-offs for starting late feeding. Overall mortality was 13 out of 56 patients (23%). One patient in each treatment arm was taken home to die; in the analysis these patients were considered as fatal cases. Baseline characteristics of the study participants according to treatment group are summarized in table 1. Except for the higher proportion of female patients in the group receiving early enteral feeding, there were no differences in baseline characteristics between the groups. Endotracheal intubation with mechanical ventilation was applied in two patients, one in each group in the intensive care unit. All other patients were managed on a general medical ward throughout their admission.

**Table 1:** Baseline characteristics of patients with cerebral malaria treated with early start of enteral feeding (on the day of admission) or late start of enteral feeding (after 60 hours of admission in adults and 36 hours in children).

Variable	Early feeding (n = 27)	Late feeding (n = 29)	P
Age (years)^a^	32 (7–70)	30 (7–75)	0.628
Male/Female patients	8/19	2/27	0.038
Weight (kg)	47 (43–52)	52 (49–55)	0.088
Height (cm)	1.51 (1.48–1.54)	1.53 (1.50–1.56)	0.318
Mid upper arm circumference (mm)	22.8 (21.6–24.1)	23.9 (23.0–24.9)	0.124
Triceps skinfold thickness (mm)	1.2 (1.1–1.4)	1.4 (1.1–1.6)	0.102
Temperature (aural, °C)	38.2 (37.7–38.7)	38.0 (37.6–38.4)	0.482
Systolic blood pressure (mmHg)	120 (113–126)	118 (113–123)	0.688
Oxygen saturation (%)^c^	93 (91–95)	93 (91–96)	0.858
Glasgow Coma Scale^b^	7 (6–9)	9 (6–10)	0.141
Hematocrit (%)	27 (24–29)	29 (26–31)	0.271
Peripheral white blood cell count (x10^3^cells/mm^3^)	14.5 (6.6–22.5)	10.4 (7.7–13.1)	0.388
Platelet count (x10^3^cells/mm^3^)	83.5 (62.7–104.3)	101,900 (74.7–129.2)	0.329
Parasitaemia (x10^3^/µl)^c^	12.8 (4.8–34.7)	7.9 (3.3–19.0)	0.516
Blood urea nitrogen (mg/dL)^c^	33 (26–41)	33 (24–44)	0.976
Venous bicarbonate level (mmol/L)	19.6 (18.0–21.2)	19.5 (17.1–21.9)	0.94
Serum base excess (mmol/L)	−4.8 (−6.9 to −2.7)	−3.7 (−5.7 to −1.7)	0.479
Glucose	136 (117–158)	141 (120–165)	0.794

Values are mean (95% CI) unless indicated otherwise;^ a^Median (interquartile range); ^b^Median (range);^ c^Geometric mean (95% confidence interval).

### Aspiration pneumonia, hypoglycaemia and coma recovery time

Aspiration pneumonia occurred in 9/27 (33% (95% CI 18–52%)) patients who started early nasogastric tube feeding, compared to none (0% (95% CI 0–14%)) in those in the late feeding group (p = 0.001, relative risk = infinity, table 2). All patients who developed aspiration pneumonia were witnessed to aspirate feed. Three of these were confirmed on chest X-ray, three died before a follow-up X-ray could be arranged, two had a normal chest X-ray at 24 hours which was not repeated and one patient had no follow-up X-rays. All patients with no radiological confirmation had clear signs of pneumonia on physical examination including fever, tachypnoea, bronchial breathing with auscultatory crackles and low peripheral oxygen saturations. All patients with aspiration pneumonia were >12 years of age. Because of limited availability, only one patient with aspiration pneumonia could receive mechanical ventilation. In-hospital case fatality rate in patients developing aspiration pneumonia was 5/9 (56%). Aspiration pneumonia became apparent in 8/9 (89%) patients within 24 hours of commencing feeds and 8/9 (89%) patients who developed pneumonia had a GCS<9 at the moment of aspiration. Gastric retention was systematically checked and occurred in 4/27 (15%) of patients receiving early enteral feeding. Despite the strict protocol withholding feeds in case of retention, 2 of these patients developed aspiration pneumonia. Vomiting occurred in 8/56 (14%) patients, but only two of these developed aspiration pneumonia. Patients with aspiration pneumonia had a lower GCS on admission (median (interquartile range) 6 (4–7) versus 8 (6–10), p = 0.037). Multiple convulsions were reported for 8 (30%) and 5 (17%) patients, respectively (p = 0.35) before enrollment. Five of the eight (62.5%) patients in the early group suffered aspiration pneumonia, two of whom had further convulsions after enrollment. There was no difference in admission oxygen saturation (geometric mean (95% CI) 92 (91–93) % versus 93 (92–94) %, p = 0.711).

**Table 2:** Disease outcome in patients with cerebral malaria treated with early start of enteral feeding (on the day of admission) or late start of enteral feeding (after 60 hours of admission in adults and 36 hours in children).

Variable	Early feeding (n = 27)	Late feeding (n = 29)	p
Aspiration pneumonia	9, 33% (18–52%)	0, 0% (0–14%)	0.001
Hypoglycaemia	0, 0% (0–15%)	1, 3% (0–19%)	1
Coma recovery time* (hours)	40 (29–51)	32 (20–48)	0.319
Sepsis	8, 30% (16–49%)	7, 24% (12–42%)	0.765
Time to sit* (hours)	72 (42–95)	55 (43–84)	0.732
Time to speak* (hours)	24 (18–44)	32 (16–44)	0.795
Time to eat* (hours)	75 (45–99)	72 (48–87)	0.523
Duration of admission* (days)	6 (5–7)	5 (5–7)	0.428
Death	9, 33% (19–52%)	6, 21% (9–39%)	0.370
Aspiration + death	5, 19% (8–37%)	0, 0% (0–14%)	0.021

Values are shown with 95% confidence intervals unless indicated otherwise; *median (interquartile range).

Blood glucose concentrations were checked every 4 hours and hypoglycemia occurred in only one patient (3.5% (95% CI 0–19%)) in the late feeding group feeding, compared to none (0% (95% CI 0–15%)) in the early group (p = 1.0, relative risk = 1). The geometric mean (95% CI) volume of feed given per patient was 2.7 (1.6–4.7) L over a median (quartiles) of 2 (1–3) days.

There was no difference in coma recovery time between the two groups (table 2).

### Secondary outcome measures

There were no differences in incidence of sepsis, times to sit, speak or eat independently, duration of admission or overall mortality between the two groups (table 2). Median (interquartile range) time to death was 1 (1–6) days. Mortality was 5/9 (56%) in patients with aspiration pneumonia, all in the early feeding group. Two out of six of those who died in the late feeding group had received nasogastric feeding. Both of these had multiorgan failure and there was no evidence of either aspiration or aspiration pneumonia.

## Discussion

Start of early enteral feeding is an established and evidence based strategy in critically ill patients in the intensive care unit. However, the evidence for this practice is obtained in well-resourced Western ICU settings, whereas the strategy is also followed in many hospitals in resource-poor settings where endotracheal intubation is not generally available. Early start of nasogastric tube feeding was routine practice in CMCH at the moment the current study was instigated. We showed that almost a third of cerebral malaria patients receiving early enteral feeding on the first day of admission developed aspiration pneumonia, whereas no patient in the control group did. Although there was no difference in overall mortality between those who received early and late enteral feeding, there was an excess mortality in those with aspiration pneumonia. Previous studies have shown that early enteral feeding can reduce mortality [22] and incidence of sepsis [3], hasten recovery [23] and shorten length of hospital stay [24], but all these studies were done in well-resourced settings, including round the clock availability of endotracheal intubation and mechanical ventilation. In the current study none of these potential benefits of early enteral feeding could be detected. The results of our study clearly illustrate that recommendations based on evidence obtained in a resource-rich setting, such as most of the ‘surviving sepsis guidelines’, should be subjected to critical appraisal before being translated to settings with limited resources [25]. Evaluating certain interventions, such as glucose control or fluid management in severe sepsis, will require a setting specific randomized clinical trial. The current study shows that a generally accepted concept, that is the benefit of early enteral feeding, is not necessarily applicable to the resource poor environment. Many clinical guidelines are based on evidence gathered in resource rich settings. A critical review of this evidence in light of the often very different facilities available in the developing world seems warranted.

Vomiting and gastric retention were not identified as risk factors for aspiration pneumonia in this study, which is in contrast with previous studies [26], [27], although recent evidence from critically ill patients indicates aspiration is also frequent in those with low gastric residual volumes [28]. Convulsions before admission were associated with aspiration, although in all patients with aspiration pneumonia, aspiration was witnessed to occur later.

In accordance with normal practice in many hospitals in the region, much of the nursing care, including administration of enteral feeding, was provided by patients’ relatives rather than trained staff. Although they were carefully instructed, and volumes of feeds were carefully monitored, adherence to the study procedures by these family members could not routinely be verified. It is possible that the incidence of aspiration may have been lower if more staff had been available to assist with care. Another shortcoming was that in this unblinded randomized study, the assessment of aspiration pneumonia was supported by positive findings on the chest X-ray in only 3/9 patients, whereas in 6/9 patients an X-ray could not be obtained. However, findings on physical examination were clearly compatible with pneumonia in all these cases and pneumonia only occurred in the early feeding group following witnessed aspiration after commencing feed thus leaving little room for alternative diagnoses.

Most patients developing aspiration pneumonia aspirated during the first 24 hours of admission, while still being unconscious, and they had a lower median GCS than those who did not aspirate. This is in agreement with other studies describing increased risk of aspiration pneumonia in patients with a GCS<9 [26], since they will be less capable of protecting the airway. Surviving patients with cerebral malaria frequently recover consciousness over the first 24–72 hours. For this reason, most of the patients in the late feeding group did not receive enteral feed as it was no longer indicated. The current study shows that enteral feeding can be safely withheld during this period, with a low incidence of hypoglycaemia and without a prolongation in the time to recovery.

In conclusion, this study provides evidence that early enteral feeding is detrimental in patients with cerebral malaria treated in resource poor settings where endotracheal intubation is not generally available. Early enteral feeding increases the risk of aspiration pneumonia and conveys no clear benefits. Evidence gathered in resource rich settings is not necessarily transferable to resource-poor settings.

## Supporting Information

Protocol S1 **Study protocol.**
(DOC)

Checklist S1 **CONSORT 2010 checklist.**
(DOC)
