# Supplementary material for: Timing of Enteral Feeding in Cerebral Malaria in Resource-Poor Settings: A Randomized Trial
Source: PLoS One. 2011 Nov 16;6(11):e27273. doi: 10.1371/journal.pone.0027273 (PMC3217943; doi:10.1371/journal.pone.0027273)
Supplement: Protocol S1 — Study protocol. (DOC) [file pone.0027273.s001.doc]

**Oxtrec reference nr. 19-08**

**Timing of Enteral Feeding in Cerebral Malaria in the Tropical Setting:**

**A Randomized Trial**

**PROTOCOL**

*Investigators*

Dr. Md. Amir Hossain^1^*, Dr. Arjen Dondorp^2,3^, Dr. Richard Maude^2,3^, Dr. Prakaykaew Charunwatthana^2^,Dr. Rasheda Samad^1^, Prof. Md. Abu Sayeed, Prof. Emran Bin Yunus^1^, Prof. Md. Ridwanur Rahman^1^, Dr. Shahena Akter^4^, Prof. Md. Gofranul Haque^5^, Prof. Md. Mahtab Uddin Hasan^5^, Dr.Badrul Alam^5^ , Prof. Nicholas Day^2,3^, Prof. M.A. Faiz^1^

1. Malaria Research Group (MRG), 1051/A, O.R Nizam Road, Mehdibag, Chittagong, Bangladesh. 2. Mahidol-Oxford Tropical Medicine Research Unit, Faculty of Tropical Medicine, Mahidol University, 420/6 Rajvithi Road, Bangkok 10400, 3. Centre for Tropical Medicine, Nuffield Department of Clinical Medicine, John Radcliffe Hospital, University of Oxford, Oxford, U.K., 4. Fatik Chari Thana Health Complex, Chittagong, Bangladesh, 5. Chittagong Medical College Hospital, Chittagong, Bangladesh. * Principal investigator. Author for correspondence: amir_hossain_ctg@yahoo.com.

*Introduction*:

Although the treatment with artesunate compared to quinine considerably reduces mortality in severe and cerebral malaria, the case fatality rate remains high at between 20% and 30%[[1]](#endnote-2). Improved supportive care can importantly further reduce mortality; a series from a well equipped intensive care unit in Paris showed a mortality of 11% in patients with severe malaria[[2]](#endnote-3). Supportive treatments include highly technical aids like renal replacement therapy and mechanical ventilation, but also easier to achieve treatments like enteral feeding. In the well equipped intensive care setting early start of enteral feeding in a wide variety of patients, including those with sepsis, is now common practice. Nutrition supplies vital cell substrates, antioxidants, vitamins, and minerals, essential for normal cell function. Studies have shown that early enteral feeding preserves the barrier function of the gut, has positive effects on immune functions, is associated with a decrease in hypermetabolism and organ failure, and reduces the chance of bacteraemia [[3]](#endnote-4) [[4]](#endnote-5) [[5]](#endnote-6) [[6]](#endnote-7) [[7]](#endnote-8) [[8]](#endnote-9). In severe malaria, patients are often poorly nourished and hypoglycaemia is a common complication, especially in patients receiving quinine. Also, bacteraemia with Salmonella species is more common in severe malaria[[9]](#endnote-10), associated with increased bacterial translocation in the gut. Enteral feeding might have a beneficial effect here. The downside of starting enteral feeding in the dependent comatose patient is the risk of aspiration pneumonia; this risk is also present in the mechanically ventilated patient[[10]](#endnote-11) [[11]](#endnote-12), although the risk is lower with the use of post-pyloric enteral feeding tubes[[12]](#endnote-13) [[13]](#endnote-14) [[14]](#endnote-15). A supine position of the patient is a risk factor[[15]](#endnote-16). Routine endotracheal intubation to protect the airway in comatose malaria patients is not a feasible option in most of the tropical countries where malaria is endemic. The use of enteral feeding through a naso-gastric (NG) tube might thus induce a much higher risk of aspiration pneumonia, outweighing the theoretical benefits.

At Chittagong Medical College Hospital (CMCH), the current practice is to start early enteral feeding through a NG-tube, with a volume of 2-4ml/kg per feed every 2 hours, avoiding 2 late night feeds, resulting in a total of 10 feeds per day for adults. For children fluid supplementation is as follows: for age 2-4years: 100 - 120ml/kg/day; 4-8yrs 90-100ml/kg/day; 8-12yrs 70-90 ≥12 yrs 60-70ml/kg/day given every 2 hours round-the-clock. In both the cases the enetral feeding volume is derived from total volume subtracting the volume given as i.v. fluids. The feeds are prepared from locally available food by blending or commercially available powder form. Although not formally assessed, aspiration pneumonia is a rather common complication of NG feeding in CMCH. Factors that can contribute include the supine position of the patient and no check for gastric retention before the next feeding. We here propose a randomized trial to compare the start of early versus late nasogastric tube feeding, with aspiration pneumonia, incidence of hypoglycaemia and coma recovery times as primary outcome measures.

Materials and Methods

*Place and Period of Study*

It is planned to study 124 consecutive patients, age ≥2 y/o, with cerebral *Plasmodium falciparum* malaria, defined as a Glasgow Coma Score (GCS) below 11or BCS <3 (for pre-verbal children) and the presence of asexual stage *P. falciparum* parasites in a peripheral blood slide, admitted to Chittagong Medical College Hospital, Chittagong, Bangladesh. Patients will be recruited from the internal medicine wards (ward 13, 14, and 16) and from the paediatric ward.

*Inclusion Criteria*

1. Cerebral falciparum malaria defined as a GCS<11or BCS <3 (for pre-verbal children), and the presence of asexual forms of *P. falciparum* in the peripheral blood smear.
2. The attending relative able and willing to give informed consent.
3. ≥2 years of age

*Exclusion Criteria*

1. Relatives unable or unwilling to give informed consent.
2. Patients who already have the features of aspiration pneumonia.
3. Pregnancy
4. Diabetic requiring insulin
5. Contraindications to enteral feeding:

-Circulatory shock

-Mechanical bowel obstruction / ileus / ischaemic colitis (redcurrant jelly stools)

-Severe diarrhea (> 6 per 24 hours), severe vomiting (> 6 per 24 hours) or severe dehydration due to either

-Pancreatitis (laboratory confirmed: serum amylase >500 U/L)

6. Known allergies to artesunate or quinine

7. Severely malnourished child (according to WHO criteria)

*Examination and Treatment*

On enrolment a full history and physical examination will be carried out. Nutritional status of the patient will be assessed by skin-fold thickness in the triceps region of the left arm and the mid upper arm circumference will be measured. Weight/height and presence of bilateral pedal edema (WHO criteria) for malnourished children will be assessed.

First line antimalarial treatment will be with intravenous artesunate, if available. Artesunate (Guilin No 2 Pharmaceutical Factory, Guangxi, Peoples’ Republic of China) will be given in a dose of 2.4 mg/kg body weight on admission, then at 12 hours, 24 hours, and thereafter once daily until oral medication can be taken reliably. Each 60 mg vial contains anhydrous artesunic acid which is dissolved initially in 1 ml 5% sodium bicarbonate and then mixed with 4 ml of 5% dextrose before injecting as a bolus into an indwelling intravenous cannula. When the patient has recovered sufficiently to take tablets, a full adult course of artemether-lumefantrine (Coartem^R^) will be given: 4 tablets twice daily for 3 days. Currently injectable artesunate is not registered yet in Bangladesh for the treatment of severe malaria. In case artesunate for injection is not available, the patient will be treated with i.v. quinine. A loading dose of 20 mg/kg will be given over 4 hours time followed by a dose of 10 mg/kg over 2 to 4 hours every 8 hours for a total of 7 days, or until the patient has recovered sufficiently to take tablets, in which case a full course of coartem will be given (see above). Patients will be managed in accordance with the World Health Organization guidelines 2006.

*Treatment allocation*

All patients will receive a NG tube. Patients will be randomized to either receive enteral feeding upon admission through the NG tube *(arm 1)*, or no feeding until able to take oral food or maximum until 60 hours (adults i.e. >12 years old) or 36 hours (children i.e. <12 years old) after admission followed by enteral feeding *(arm 2)*. The feed used in the study will be Revit-R/Renovit(patient with renal insufficiency) (Fasska SA, Louvain la Neuve, Belgium. The preparation comes as a powder, which will be dissolve in bottled water according to the instructions of the manufacturer (220g per liter water which will provide 1 kcal/ml) The feeding protocol is as follows:

Check position of NG tube before start of the first feed with a large syringe and before subsequent feeds by checking the position of a mark drawn on the tube at the time of insertion. Position the patient in the head tilt position (head 15° above horizontal). Start NG feeding with 2-4 ml/kg feeding every 2 hours in adult 10 feeds per day; omitting 2 late night feeds, this will deliver1000-2000kCal per 24 hours for adults weighing 50 kg. For children, the volume of enteral feeding will be calculated from the total amount of fluids to be given per 24 hours as follows: for age 2-4years: 100 - 120ml/kg/day; 4-8yrs 90-100ml/kg/day; 8-12yrs 70-90 ≥12 60-70ml/kg/day. The enteral feeding volume is derived from this total volume subtracting the volume given as i.v. The total amount of enteral feeding in children is divided in 12 feeds, given every 2 hours round-the-clock

Gastric retention will be measured just before the next feeding. Gastric retention is defined as> 4 ml/kg in adult and >2/3^rd^ of the 2 hours previous feed in children. If gastric retention is present: feeding is discontinued and prokinetic drugs domperidon suppositories are added for adults domperidon 20 mg suppositories, every 8 hours; for children: domperidone suppository 15mg 1/2 suppository up to 5 years & 1 suppository beyond 5 years. In adults retention is reassessed 4 hourly. If the retained volume is <4 ml/kg, discard this volume, and feeds are continued every 2 hours as before. In children retention reassessed 2hourly and if the retention is < 2/3^rd^ of the previous 2 hours feed this volume discard it, and feeds are continued every 2 hours as before.

Adult patients in the control arm (delayed start of enteral feeding) will receive 50-60ml 5% DS (glucose-saline)/kg/24 hours. The energy contents the infusion fluid containing 50-60g dextrose per liter around 200 kCal per liter, so that the energy given is 10-12 kCal/kg/ 24 hours, or 500-600 kCal for the average 50 kg patient. Paediatric patients in the control arm (arm 2) will receive 10% Dextrose in half strength saline (10% dextrose in 0.45% NaCl) in the volumes as above. This contains 400kCal/L, so that daily total calories delivered to the children not receiving enteral feeding will be for 2-4 y/o: 40-48 kCal/kg, for 4-8 y/o: 36-40 kCal/kg and for 8-12 y/o: 24-28 kCal/kg.

Family members will be instructed in the techniques for checking NG tube position using a pre-drawn mark, checking for gastric retention and giving NG feeds.

*General care measures for the patient with cerebral malaria will include:*

Head tilt position (15° to 30°). Recording of GCS, blood pressure, respiratory rate, temperature, and oxygen saturation (pulse-oxymetry) every **4 hours.** Maintenance of an open airway, if needed, with a Guedel. Nursing on the side with turning every **2 hours** by the patient’s relatives to prevent bed sores. Catheterization of the bladder (Foley catheter) and recording of fluid input/output. Eye care with moisturizing eye ointment in the case of open eyes. Anti-pyretics: in case of fever, paracetamol 1g for adults and 15 mg/kg for children under 12 years will be given unless contra-indicated (liver failure). Once conscious and taking oral feed patients should be followed-up once daily.

*Investigations*

On admission a 10 ml blood sample will be taken for parasitaemia, complete blood count (CBC), including leukocyte differentiation and percentage of band form neutrophils, venous pH*, bicarbonate* and blood standardized base excess*, glucose*, electrolytes*, blood urea nitrogen*. (Parameters marked with * will be measured by i-STAT, using the EC8+ cartridges).

Peripheral blood parasitaemia and staging in thin film will be assessed on admission, followed by 12 hourly sampling until complete parasite clearance, defined by 2 consecutive negative peripheral blood slides.

Blood glucose will be checked 4 hourly until the patient regains consciousness. A blood glucose level below ≤2.8 mmol/l (50mg/dl) will be treated with 10% intravenous glucose (4 ml/kg body weight).

Blood count (i.e. total and differential white cell count, platelet count, haemoglobin and haematocrit), biochemistry and blood cultures will be repeated on indication. In particular, a blood count will be repeated when aspiration pneumonia or sepsis is suspected.

*Aspiration pneumonia*

Aspiration pneumonia will be diagnosed clinically from history and chest examination (fever, respiratory distress, auscultatory crackles, oxygen saturation), and confirmed by chest X-ray (CXR). All patients will have a CXR upon admission. If possible, a CXR will be done in the radiology department of CMCH. If the patient is not transportable, a bed side CXR will be performed. The CXR will be repeated upon indication and 3 days after the first one for follow-up. Treatment will be with antibiotics (intravenous ceftriaxone in combination with metronidazole). Oxygen will be given by nasal cannula or face mask where indicated. In the case of severe respiratory insufficiency, a concerted effort will be made to transfer the patient to the intensive care unit of CMCH for mechanical ventilation.

Analysis

*Primary outcome measures include:*

1. Incidence of aspiration pneumonia.
2. Incidence of hypoglycaemia (<2.2 mmol/L).
3. Coma recovery time. The coma recovery time is defined as the time till a Glasgow Coma Score of 15/15 (BCS 5/5 in preverbal children).

*Secondary outcome measures include:*

1. Incidence of sepsis.

Sepsis is defined as the presence of infection (other than malaria) in combination with systemic inflammatory response syndrome (SIRS) as indicated by ≥ 3 of the following criteria: prolonged fever i.e. axillary temperature ≥ 38ºC or core temperature of ≤ 36ºC; a heart rate of ≥ 90 beats/min; a respiratory rate of ≥ 20 breaths/min (up to 5yrs ≥40) or the use of mechanical ventilation for an acute respiratory process; a white-cell count of ≥ 12 x 10^9^/l or ≤ 4 x 10^9^/l, or a differential count showing > 10% immature neutrophils.

2. Time to sit independently, which will be assessed daily.

3. Time to speak independently, which will be assessed daily.

4. Time to eat independently, which will be assessed daily.

5. Total duration (days) of admission in the hospital.

6. Survival. In hospital mortality will be recorded.

Sample size calculation

To detect an increase in incidence in aspiration pneumonia from 10% to 30%, a sample size of 124 (62 in each treatment group) is required. After this pilot study the data will be analysed. If early enteral feeding appears to be promising regarding a decrease in hypoglycaemia or a shortening of coma recovery times, not at the expense of a significant increase in aspiration pneumonia, a larger follow-up trial will be planned.

Randomisation

Randomisation will be balanced in blocks of 20 by computerized method using STATA statistical software package.

Safety

Current practice in CMCH is to start on the day of admission with enteral feeding. However, if the current study shows a significant increase in the incidence of aspiration pneumonia in the group receiving early enteral feeding, randomization will be stopped, and patients will only receive enteral feeding after regaining consciousness, or 60 hours after admission. Since aspiration pneumonia is an identified risk, interim analyses will be performed after every 50 patients included. Interim data will be analysed by the Data Safety Monitoring Committee.

Benefits of the study

There is currently equipoise about the benefit of the start of early enteral feeding in patients with cerebral malaria in the tropical setting. Theoretical advantages (prevention of hypoglycaemia, restoration of immune function, etc) are countered by the increased risk of aspiration pneumonia. The study has a clear practical goal to guide this important part of the treatment of patients with cerebral malaria.

Finance

Costs involved in the study will be financed by the Wellcome Trust of Great Britain

References

1. SEAQUAMAT study group. Artesunate versus quinine for treatment of severe falciparum malaria: a randomised trial. Lancet 2005; 366: 717-725 [↑](#endnote-ref-2)
2. Bruneel F, Hocqueloux L, Alberti C et al.. The clinical spectrum of severe imported falciparum malaria in the intensive care unit: report of 188 cases in adults. Am J Respir Crit Care Med 2003; 167:684-9 [↑](#endnote-ref-3)
3. Moore FA, Moore EE, Jones TN, McCrosky BL, Peterson VM. TEN versus TPN following major abdominal trauma reduced septic morbidity. J Trauma 1989; 29: 916-923. [↑](#endnote-ref-4)
4. Chiarelli A, Enzi G, Casadei A, Baggio B, Valerio A, Mazzoleni F. Very early enteral nutrition supplementation in burned patients. Am J Clin Nutr 1990;51:1035-1039. [↑](#endnote-ref-5)
5. Beier-Hogersen R, Boesby S. Influence of postoperative enteral nutrition on postsurgical infections. Gut 1996;39:833-835. [↑](#endnote-ref-6)
6. Kompan L, Krežar R, Gadžijev E, Prošek M. Effects of early enteral nutrition on intestinal permeability and the development of multiple organ failure after multiple injury. Intensive Care Med 1999; 25: 157-161. [↑](#endnote-ref-7)
7. Marik PE, Zaloga GP. Early enteral nutrition in acutely ill patients: A systematic review. Crit Care Med 2001;29:2264-2270. [↑](#endnote-ref-8)
8. Nelson JL, Foley-Nelson TL, Gianotti L. Caloric intake and bacterial translocation

   following burn trauma in guinea pigs. Nutrition 1999. [↑](#endnote-ref-9)
9. Walsh AL, Phiri AJ, Graham SM, Molyneux EM, Molyneux ME. Bacteremia in febrile Malawian children: clinical and microbiologic features. Pediatr Infect Dis J. 2000;19:312-8. [↑](#endnote-ref-10)
10. Jacobs S, Chang RW, Lee B, Bartlett FW. Continuous enteral feeding: a major cause of

    pneumonia among ventilated intensive care unit patients. JPEN J Parenter Enteral Nutr 1990;

    14(4):353-356. [↑](#endnote-ref-11)
11. Bosscha K, Nieuwenhuijs VB, Vos A, Samsom M, Roelofs JM, Akkermans LM.

    Gastrointestinal motility and gastric tube feeding in mechanically ventilated patients. Crit

    Care Med 1998; 26(9):1510-1517. [↑](#endnote-ref-12)
12. Kearns PJ, Chin D, Mueller L, Wallace K, Jensen WA, Kirsch CM. The incidence of

    ventilator-associated pneumonia and success in nutrient delivery with gastric versus small

    intestinal feeding: a randomized clinical trial. Crit Care Med 2000; 28(6):1742-1746. [↑](#endnote-ref-13)
13. Heyland DK, Drover JW, MacDonald S, Novak F, Lam M. Effect of postpyloric feeding on

    gastroesophageal regurgitation and pulmonary microaspiration: results of a randomized

    controlled trial. Crit Care Med 2001;29(8):1495-1501. [↑](#endnote-ref-14)
14. Davies AR, Froomes PRA, French CJ, Bellomo R, Gutteridge GA, Nyulasi I, Walker R,

    Sewell RB. Randomized comparison of nasojejunal and nasogastric feeding in critically ill

    patients. Crit Care Med 2002;30(3):586-590. [↑](#endnote-ref-15)
15. Drakulovic MB, Torres A, Bauer TT, Nicolas JM, Nogue S, Ferrer M. Supine body position as a risk factor for nosocomial pneumonia in mechanically ventilated patients: a randomized trial. Lancet 1999; 354(9193):1851-185. [↑](#endnote-ref-16)
